# Ferroptosis and endoplasmic reticulum stress in rheumatoid arthritis

**DOI:** 10.3389/fimmu.2024.1438803

**Published:** 2024-07-15

**Authors:** Qin Ao, Huan Hu, Ying Huang

**Affiliations:** ^1^ Guizhou Universisity of Traditional Chinese Medicine, Guiyang, China; ^2^ Department of Rheumatology and Immunology, The Affiliated Hospital of Guizhou Medical Universisity, Guiyang, China; ^3^ Center for General Practice Medicine, Department of Rheumatology and Immunology, Zhejiang Provincial People’s Hospital (Affiliated People’s Hospital, Hangzhou Medical College), Hangzhou, China

**Keywords:** ferroptosis, endoplasmic reticulum stress, rheumatoid arthritis, lipid peroxidation, reactive oxygen species, unfolded protein response

## Abstract

Ferroptosis is an iron-dependent mode of cell death distinct from apoptosis and necrosis. Its mechanisms mainly involve disordered iron metabolism, lipid peroxide deposition, and an imbalance of the antioxidant system. The endoplasmic reticulum is an organelle responsible for protein folding, lipid metabolism, and Ca^2+^ regulation in cells. It can be induced to undergo endoplasmic reticulum stress in response to inflammation, oxidative stress, and hypoxia, thereby regulating intracellular environmental homeostasis through unfolded protein responses. It has been reported that ferroptosis and endoplasmic reticulum stress (ERS) have an interaction pathway and jointly regulate cell survival and death. Both have also been reported separately in rheumatoid arthritis (RA) mechanism studies. However, studies on the correlation between ferroptosis and ERS in RA have not been reported so far. Therefore, this paper reviews the current status of studies and the potential correlation between ferroptosis and ERS in RA, aiming to provide a research reference for developing treatments for RA.

## 1 Introduction

Rheumatoid arthritis (RA), a chronic autoimmune disease, is characterized by synovial proliferation and progressive bone destruction. Its pathogenesis involves tumor-like proliferation of synovial fibroblasts (FLS), local infiltration of immune cells, and the release of inflammatory cytokines that exacerbate joint inflammation and bone destruction (1, 2). Therefore, inhibiting synovial cell inflammatory proliferation and regulating the immune-inflammatory response are the main strategies for current RA treatment (3). Disease-modifying anti-rheumatic drugs (DMARDs) are routinely used for RA; however, adverse effects, drug resistance, and poor efficacy are often observed in initial treatment. Thus, further understanding of the pathogenesis of RA is urgently needed, and finding safer and more effective therapeutic targets is crucial.

Ferroptosis is a mode of iron-dependent cell death that is morphologically and biochemically distinct from apoptosis, necrosis, and pyroptosis (4, 5). It was discovered by Dion’s team in 2012 when they studied the mechanism of Erastin against tumor RAS mutations and named ferroptosis. The main features of ferroptosis are abnormal iron accumulation, lipid peroxidation, and dysregulation of the antioxidant system (6). It has been reported for many years that iron metabolism is abnormal in RA, with iron content in the synovial membrane and synovial fluid significantly higher than in peripheral blood. Additionally, the serum soluble transferrin receptor (sTFR) level is positively correlated with RA disease activity (7, 8). The localized accumulation of iron ions in the joints can promote the production of large amounts of reactive oxygen species (ROS). Coupled with the dysregulation of the antioxidant system, this can lead to bone destruction, abnormal proliferation of RA-FLS, and the massive production of inflammatory mediators (9).

The endoplasmic reticulum (ER), an important organelle in eukaryotic cells, is involved in protein synthesis, folding, structural maturation, lipid metabolism, and Ca^2+^ regulation. Inflammation, oxidative stress, Ca^2+^ dysregulation, and hypoxia can induce cellular endoplasmic reticulum stress (ERS) by overloading the ER’s protein folding mechanism (10). This subsequently stimulates the cell to initiate the unfolded protein response (UPR) to regulate the ER’s protein folding capacity (11). However, sustained and high-intensity ERS can lead to cell death (12). RA, an autoimmune disease, primarily involves the activation of immune cells, which produce large amounts of autoantibodies that attack normal tissues and trigger an inflammatory cascade through the release of inflammatory mediators, exacerbating disease progression. This process involves the endoplasmic reticulum. The inflammatory milieu of RA activates ERS, while protein synthesis is accelerated through the UPR, enabling RA-FLSs to adapt to and tolerate the milieu, exhibiting strong resistance to ERS-induced apoptosis. This may also be responsible for the tumor-like transformation or dedifferentiation of RA-FLS (13).

Some studies have found that ferroptosis and ERS share a common pathway, especially in tumor diseases, and disease trends can be altered by controlling the ERS or ferroptosis pathway (14–17). However, there is no description of the mutual crosstalk between ERS and ferroptosis in the current studies of RA pathogenesis. Therefore, this paper summarizes the roles of ferroptosis and ERS in RA and their potential relevance in the current research status, which provides a direction for exploring the mechanism of RA and new therapeutic targets.

## 2 Ferroptosis in RA

With the study of RA disease mechanisms, it has been found that the aberrant activation of RA-FLSs, destruction of articular cartilage, overactivation of immune cells, and sustained expression of inflammatory factors are associated with ferroptosis. Targeting RA ferroptosis as a potential therapeutic strategy (18).

### 2.1 Iron metabolism in RA

Iron plays an important role in human growth and development, immune system function, and energy metabolism (19). Abnormalities in iron metabolism can affect gene regulation, enzymatic reactions, redox reactions, and other important processes (20, 21). There are two forms of iron in the human body: Fe^2+^ and Fe^3+^. Circulating iron (Fe^3+^) binds to transferrin (Tf), which then binds to specific transferrin receptor 1 (TFR1) and transfers to the cell. Inside the cell, it is deoxygenated and reduced to Fe^2+^ by iron oxide reductase. In the cytoplasm, Fe^2+^ is mainly bound to ferritin to form storage iron, with a small portion stored in the cytoplasm’s labile iron pool (LIP) (21). Due to the instability and high oxidizability of Fe^2+^, it can generate hydroxyl radicals through the Fenton reaction. These radicals can directly catalyze the oxidation of polyunsaturated fatty acid (PUFA) in the cytosol and plasma membrane under the action of lipoxygenase (LOX), generating a large amount of lipid ROS. This process causes oxidative damage to lipid membranes, proteins, and DNA, ultimately leading to cell death (22). Among the three major mechanistic pathways of ferroptosis, the abnormal accumulation of iron is the initial part of its cascade reaction. The large accumulation of Fe^2+^ in LIP increases the sensitivity of cells to ferroptosis.

Abnormal iron metabolism in RA is closely related to disease progression. A study showed that serum sTFR levels were significantly higher and serum iron levels were significantly lower in patients with RA compared to healthy controls. sTFR was also found to be significantly and positively correlated with parameters of inflammatory activity and autoimmune disease (23). Later, Wu J et al. (24)found that iron levels in synovial fluid and lipid peroxides in the synovium were significantly higher in RA patients with high disease activity than in those with moderate disease activity, while iron levels in peripheral blood were decreased. This suggests that iron distribution and metabolism are disordered in RA. Iron overload can inhibit osteoblast activity and promote osteoclast differentiation to a certain extent (25).It inhibits precursor cells (MC3T3-E1) from differentiating into osteoblasts by promoting ROS production, blocking the phosphatidylinositol 3-kinase (PI3K/AKT) and Jak/Stat3 signaling pathways, and activating the p38 MAPK, which induces G1-phase blockade and autophagy in osteogenic MC3T3-E1 cells (26). Meanwhile, iron overload also induces osteoclast differentiation and accelerates articular bone destruction by activating JNK, ERK, and NF-kB signaling pathways (27, 28). As a major effector cell of RA arthropathy, elevated ROS and lipid oxidation triggered by iron overload can also lead to the abnormal proliferation of RA-FLSs (24).

Iron, as a regulator of the immune response, affects the phagocytosis of monocytes and macrophages. Iron overload alters the number and viability of T-lymphocyte subpopulations and the distribution of lymphocytes in different compartments of the immune system (29). Under the same ferrous stimulus, anti-inflammatory M2-like macrophages undergo significant ferroptosis, while pro-inflammatory M1-like macrophages experience less ferroptosis. Additionally, iron overload leads to the release of high mobility group protein 1 (HMGB1) in the damage-associated molecular patterns (DAMPs) released from cells. HMGB1 can act as an adjuvant to activate the immune system and promote the secretion of tumor necrosis factor-α (TNF-α) and interleukin-6 (IL-6) by macrophages. The release of inflammatory mediators increases the uptake of iron by monocytes, increasing their sensitivity to ferroptosis (30). Iron overload can also promote synovial and vascular opacification growth and aggravate the progression of synovitis by regulating *C-myc* and *Mdn2* gene expression (31).

In relation to the treatment of RA, it has been found that the iron ion chelator desferrioxamine (DFO) has been used to prevent synovial damage and improve RA anemia (32). Additionally, herbal components such as Icariin, Quercetin, and Galangin have also been shown to chelate iron ions and regulate iron metabolism, thereby reducing oxidative damage and immune dysfunction caused by iron overload (28, 33).

### 2.2 Lipid peroxidation in RA

Lipid peroxidation is central to ferroptosis. The diallyl hydrogen of PUFA has low bond dissociation energy, making it prone to lipid peroxidation (34). However, free PUFA is not directly conducive to ferroptosis. It is first converted into acyl-CoA (CoA) through the catalytic action of CoA synthase long-chain family (ACSL) located in the mitochondria and the ER. CoA is then inserted into membrane phospholipids under the catalysis of lysophosphatidylcholine acyltransferase 3 (LPCAT3) and combined with phosphatidylethanolamine (PE) to form polyunsaturated fatty acid phospholipids (PUFA-PE). PUFA-PE generates lipid peroxides mediated by LOX and produces ROS through interaction with Fe^2+^, which then extracts hydrogen from adjacent acyl chains in the lipid membrane environment, propagating the lipid peroxidation process (35).

**Figure 1:**
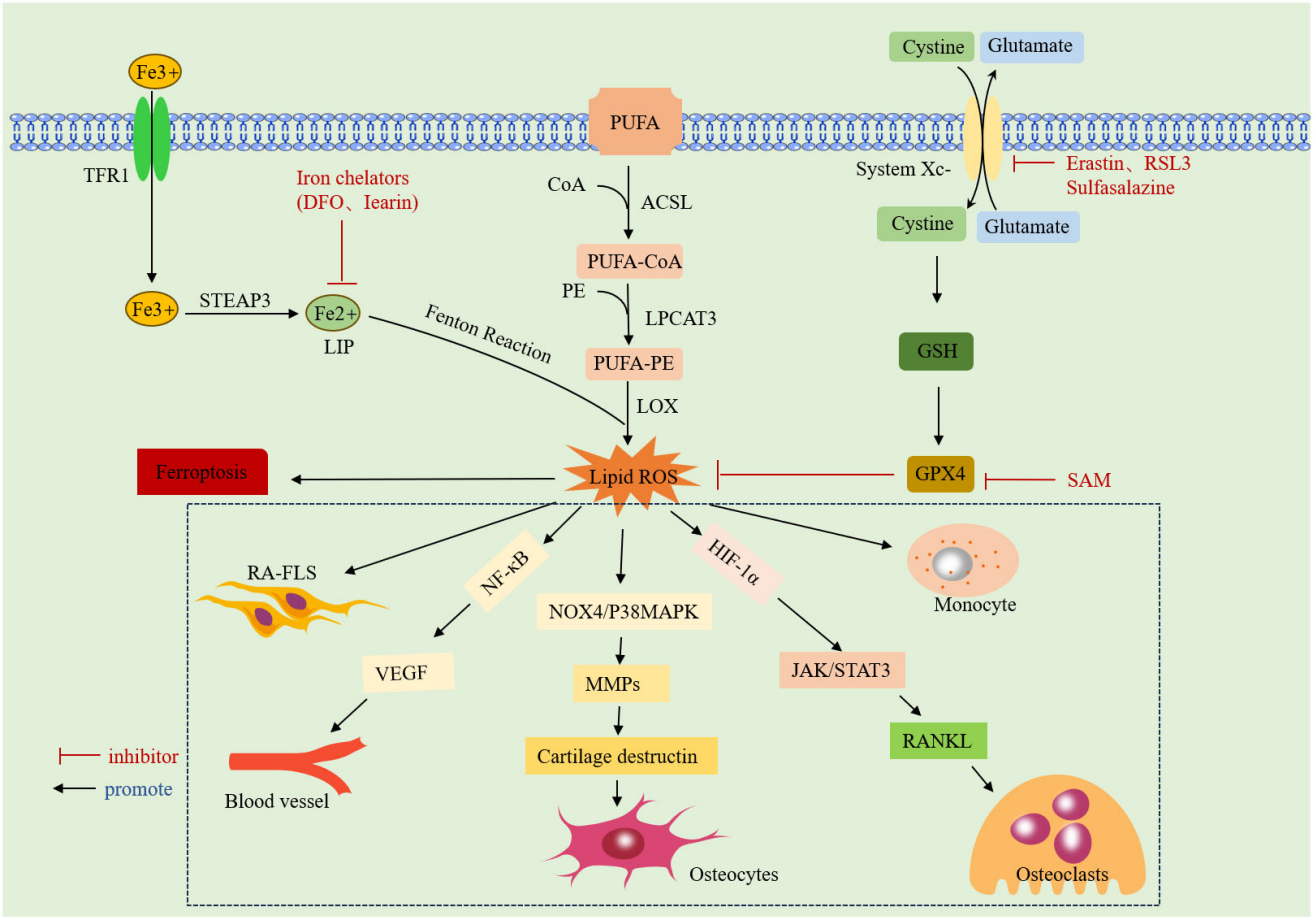
Mechanism of ferroptosis: it due to iron overload-mediated lipid peroxidation and antioxidant dysregulation. Specific introduction to the mechanism of action of ROS on RA.TFR1: transferrin receptor protein 1;STEAP3:lysophosphatidylcholine acyltransferase 3;DFO: deferoxamine;LIP:labile iron pool;CoA:acyl coenzyme A;ACSL: CoA synthase long-chain family;LPCAT3:lysophosphatidylcholine acyltransferase 3;PUFA:polyunsaturated fatty acid;PE:phosphatidylethanolamine;LOX:lipoxygenase;ROS:reactive oxygen species;System Xc-:cystine/glutamate counter transporter protein; RSL3: An inhibitor of GPX4;GSH:glutathione;GPX4: glutathione peroxidase 4; SAM:S-adenosylmethionine;HIF-1a:hypoxia inducible factor 1a;VEGF:vascular endothelial growth factor.NF-κB: nuclear factor kappa B; MMPs: matrix metalloproteinase; RANKL: receptor activator of nuclear factor-κ B ligand.

ROS interfere with various biological processes *in vivo*, including signaling, gene expression, and cellular homeostasis (36). Earlier studies by Blake DR, Roberts D, et al. found that intravenous use of iron dextrose exacerbated synovitis in RA, possibly because iron dextrose worsens synovial inflammation by promoting lipid peroxidation (37, 38). The accumulation of ROS in RA not only promotes cellular ferroptosis but also exhibits different effects on various cells. Excessive ROS are detrimental to non-synovial fibroblasts (N-FLSs); they can promote chondrocyte death by activating the NOX4/p38 MAPK pathway, inducing the expression of matrix metalloproteinases (MMPs), and degrading the cartilage matrix (39). However, ROS accumulation promotes aberrant proliferation, migration, and the release of inflammatory factors in RA-FLSs, exacerbating arthropathy (40).Additionally, ROS can promote the receptor activator of nuclear factor-κB ligand (RANKL) expression and induce osteoclast differentiation by activating the Janus kinase (JAK)2/STAT3 pathway, directly or indirectly, through upregulation of hypoxia-inducible factor-1 (HIF-1a) expression (41). They also participate in key pathological processes, such as angiogenesis and proliferation, by activating the NF-kB pathway to transcribe the vascular endothelial growth factor (VEGF) gene (42). In immune cells, ROS mediate neutrophil formation of neutrophil extracellular traps (NETs) to drive inflammatory responses (43). An overview of the mechanisms of ferroptosis and RA is given in (**Figure 1**).

### 2.3 Lipid antioxidant system in RA

When lipid peroxides accumulate, cells can resist oxidation and prevent ferroptosis through various regulatory mechanisms, the most important of which is the antioxidant regulatory system composed of the cystine/glutamate antitransporter (System Xc-) and phospholipid hydroperoxidase glutathione peroxidase 4 (GPX4) (6, 44). System Xc- is an amino acid transporter located on the cell membrane, composed of member 11 of the light chain solute carrier family (SLC7A11, xCT) and member 2 of the heavy chain solute carrier family (SLC3A2), which regulates the exchange of glutamate (Glu) and extracellular cystine (Cys) in cells. Cys is reduced to cysteine as a substrate for the synthesis of glutathione (GSH) (45). By blocking System Xc- or blocking glutamate metabolic pathways, more ROS-induced lipid peroxidation and ferroptosis can be triggered (46). Therefore, GPX4/SLC7A11 is considered a key regulator of ferroptosis and a classic pathway of ferroptosis.

With the progress of research, in addition to the GPX4 antioxidant pathway, three other new pathways unrelated to GPX4 in ferroptosis have been identified: ferroptosis inhibition protein 1 (FSP1)/CoQ10, GTP cyclic hydrolase 1 (GCH1)/tetrahydrobiopterin (BH4), and dihydroorotate dehydrogenase (DHODH). These pathways reduce lipid peroxidation, remodel lipid membranes, and prevent cell ferroptosis through direct or indirect means (47).

Compared to healthy individuals, RA patients exhibit lower expression of ACSL4 and increased levels of ferritin heavy chain 1 (FTH1), GPX4, and SLC7A11 in the synovium and FLS. Although both oxidized and antioxidant levels of lipids are higher in RA-FLS than in healthy controls, the increase in antioxidant levels is slightly higher than that of oxidation, indicating that RA-FLS is resistant to ferroptosis (48). Additionally, GPX4 is involved in the regulation of immune cell function; it is associated with B-cell development and function, and GPX4 deficiency inhibits IgM antibody production (49). Furthermore, T-cell activation is dependent on Cys, which is supplied to T-cells by the Xc- system, and overexpression of FSP1 or GPX4 protects CD8^+^ T-cells from ferroptosis (50, 51).

In studying TNF-α, an important inflammatory mediator in the pathogenesis of RA, it was found that small doses of TNF-α could enhance Cys uptake and GSH synthesis by activating the NF-κB pathway. This upregulated the functional subunits of SLC7A11 and System Xc-, protecting RA-FLSs from oxidative stress and excess iron, thereby inhibiting ferroptosis. However, this process could be blocked by a low dose of imidazole ketone erastin (IKE) in combination with the TNF antagonist etanercept (24). In contrast, treatment with IL-6, another important inflammatory mediator, increased intracellular iron levels and decreased SLC40A1 and ferritin expression, but did not alter GSH expression, making RA-FLSs more susceptible to ferroptosis. In other studies targeting ferroptosis, Ling H et al. (48)found that glycine enhanced ferroptosis via S-Adenosylmethionine(SAM) mediated GPX4 promoter methylation and ferritin decrease. The ferroptosis inducer RSL3 can induce ferroptosis by decreasing the expression of SLC2A3 and affecting the glycolytic metabolism of RA-FLS (52). Additionally, the active peptide G1dP3 promotes RA-FLS ferroptosis through a p53/SLC7A11 axis-dependent mechanism (53). Sulfasalazine, an important biological agent, was found to inhibit System Xc- (54). Beyond the classical ferroptosis antioxidant pathways, the CoQ10 pathway, which is unrelated to GPX4, was found to act as a ROS scavenger and anti-inflammatory substance. This pathway could regulate Th17 differentiation and IL-17 secretion via the STAT3 pathway, thereby inhibiting arthritis progression in CIA mice (17). Clinical trials targeting key molecules of ferroptisis is given in (**Table 1**).

**Table 1:** Clinical trials targeting key molecules of ferroptisis.

Target Name	Interventions	Conditions or Disease	ClinicalTrials.gov ID or Chinese Clinical Trial Registry	Sponsor	Primary outcomes	Phase
**Iron**	Deferiprone/Placebo	Dementia	NCT03234686	Neuroscience Trials Australia	Efficacy of Deferiprone	Phase2
	Deferiprone/ Placebo	Friedreich’s Ataxia	NCT00530127	ApoPharma	The occurrence of adverse events	Phase2
	Deferiprone	Myelodysplastic syndrome	NCT02477631	Sheba Medical Center	To evaluate ROS	Phase2
	Deferiprone	HIV	NCT02191657	ApoPharma	Adverse events	Phase1
	Deferiprone	Renal Impairment	NCT01770652	ApoPharma	Serum Deferiprone and Deferiprone 3-O-glucuronide of Cmax,Tmax,AUC0-∞,T1/2,Ae24,Fe24	Phase4
	Deferiprone/ Placebo	Parkinson’s Disease	NCT01539837	Imperial College London	Number of Serious Adverse Events	Phase2
	Deferiprone	Hepatic Impairment	NCT01767103	ApoPharma	Serum Deferiprone and Deferiprone 3-O-glucuronide of Cmax, Tmax, AUC0-∞,T1/2,CumAe,Fe%	Phase4
	Deferiprone/ Placebo	Acute Myocardial Infarction	NCT05604131	Rohan Dharmakumar	Treatment Efficacy	Phase2
	Deferasirox/ Deferiprone	Thalassemia	NCT01709032	Children’s Hospital of Philadelphia	Number of Improvement in Liver Iron Concentration	Phase2
	Deferiprone/ Acetylcysteine/Sitagliptin and Metformin	Diabetes Mellitus	NCT02882477	Hadassah Medical Organization	1.OGTT and IVGTT tests 2. Platelet function test 3. Nerve conduction velocity in VEP 4. HBA1C level 5.daily glucose level	Phase3
	Deferiprone/ Desferrioxamine	Hemochromatosis	NCT00350662	Lipomed	Clinical efficacy	Phase3
	Deferoxamine	Diabetes	NCT03085771	Karolinska University Hospital	Endothelial precursor cell account (EPC)	Phase2
	Deferoxamine	COVID-19	NCT04333550	Kermanshah University of Medical Sciences	Mortality rate	Phase2
	Deferoxamine	Leptomeningeal Metastases	NCT05184816	Memorial Sloan Kettering Cancer Center	Frequency of dose-limiting toxicities (DLTs)	Phase1
	Deferoxamine	Triple Negative Breast Cancer	NCT05300958	Sun Yat-sen University	Objective Response Rate (ORR)	Phase2
	Deferoxamine	Ischemic Stroke	NCT00777140	Germans Trias i Pujol Hospital	Clinical and Analytical Adverse Events	Phase2
	Deferoxamine/TACE	Hepatocellular Carcinoma	NCT03652467	Jinan Military General Hospital	Progression Free Survival (PFS)	Phase1
**GPX4**	NO-drugs	Diabetic Nephropathy	NCT01810822	University of Sao Paulo General Hospital	Albumin to Creatinine Ratio	No-phase
**SLC7A11**	SXC-2023 /Placebo	Trichotillomania (TTM)	NCT03797521	Promentis Pharmaceuticals, Inc.	The incidence of treatment-emergent adverse events	Phase2
**SLC3A2**	IGN523	Acute myeloid leukemia (AML)	NCT02040506	Igenica Biotherapeutics, Inc.	Incidence of adverse events	Phase2
**Glu**	VitaminE/ Memantine	Alzheimer’s Disease	NCT00235716	US Department of Veterans Affairs	1. ADCS/ADL; 2. Mini-Mental State; 3. ADAS-cog; 4. Neuropsychiatric Inventory; 5. Activity Survey	Phase3
**ACSL4**	CAFexosomes	Pancreatic cancer	ChiCTR2200061320	Tongji Hospital Affiliated to Tongji University	Mechanisms of chemotherapy resistance in pancreatic cancer	Phase0

## 3 ERS in RA

**Figure 2:**
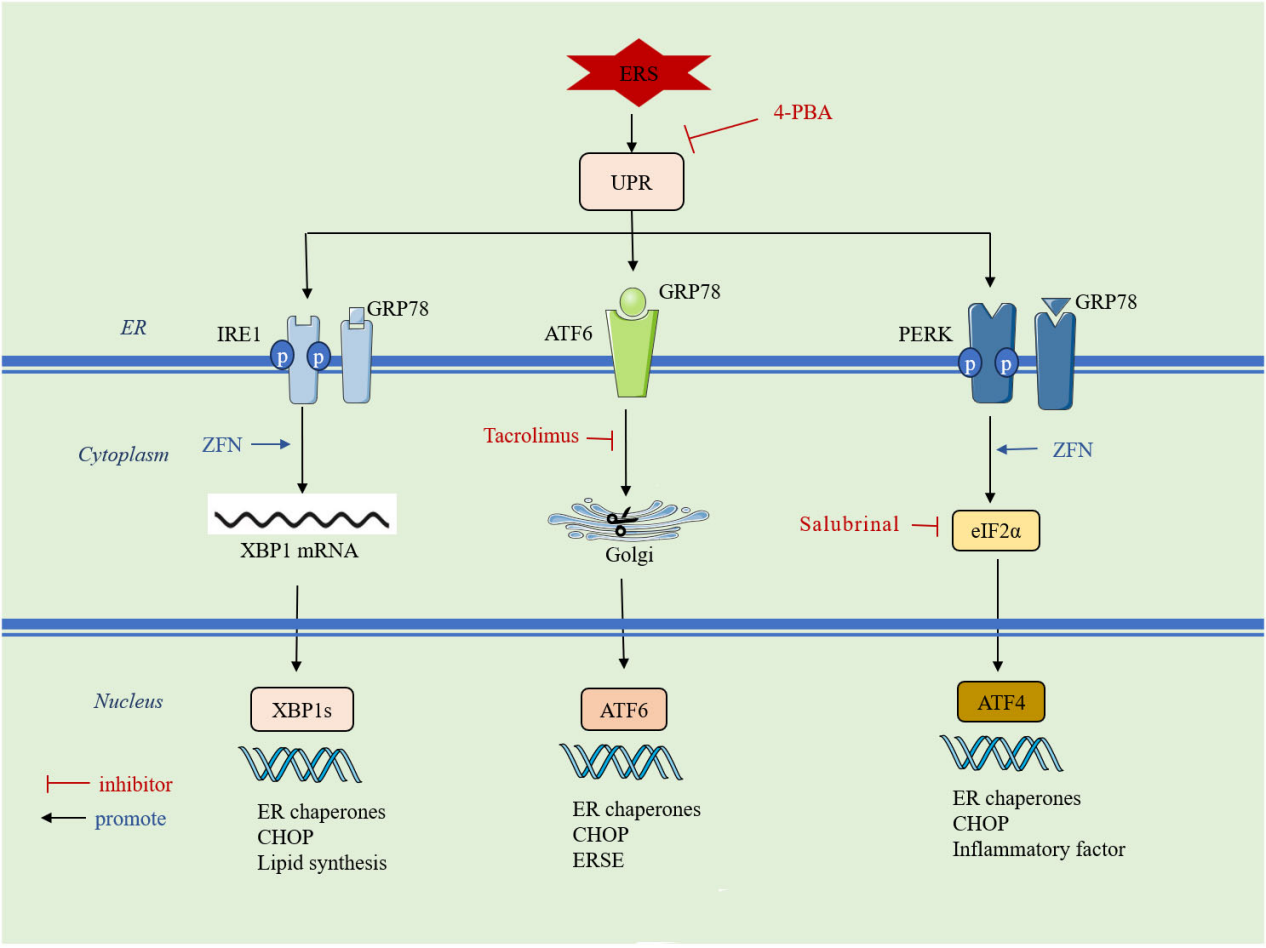
ERS initiates the UPR: it is mainly mediated by three signaling pathways, PERK, IRE1 and ATF6. ERS: endoplasmic reticulum stress; UPR: unfolded protein response; 4-PBA: 4-phenylbutyric acid; GRP78: glucose-regulated protein 78; IRE1: inositol requirement protein 1; PERK: protein kinase RNA-like ER kinase;ATF: activating transcription factor; ZFN: zinc ferrate nanoparticles;eIF2α: eukaryotic translation initiation factor 2α; XBP1: homeostasis transcription factor X-box protein 1; CHOP: C/EBP-homologous protein;ERSE:endoplasmic reticulum stress element.

Cells stimulated by internal and external injury factors can induce ERS and initiate the UPR. The UPR signaling pathway is mainly mediated by protein kinase RNA-like ER kinase (PERK), inositol-requiring enzyme 1 (IRE1), and activating transcription factor 6 (ATF6). In a physiological state, PERK, IRE1, and ATF6 are non-covalently bonded to glucose-regulated protein 78 (GRP78). When ERS occurs, GRP78 binds to misfolded proteins and dissociates from PERK, IRE1, and ATF6, activating downstream signaling pathways to inhibit DNA transcription and translation, prevent protein synthesis, and accelerate the degradation of aberrant proteins, thus regulating the adaptation of the intracellular environment. However, UPR can also activate downstream apoptotic signaling pathways and promote apoptosis when the damage is persistent and severe or when the extent of the crosstalk network exceeds the resolution capacity of ER loading aberrations (55). An overview of the mechanisms of ERS is given in (**Figure 2**).

GRP78-specific antibodies were found in up to 63% of RA patients, 7% of patients with other rheumatoid diseases, and only 1% of healthy controls (56). ERS-related genes were highly expressed in RA-FLS and synovial macrophages. *In vitro* experiments revealed that in synovial cells, TNF and IL-1β increased GRP78 expression. Inhibition of GRP78 expression using siRNA abrogated TNF- or TGF-β-induced proliferation of FLS and up-regulation of cell cycle protein D1, thereby promoting cell death. Elevated GRP78 levels, in turn, lead to synovial proliferation, which promotes further cytokine secretion, creating a vicious cycle. In the same study, it was also found that GRP78 can directly control VEGF 165-induced angiogenesis (57). Recently, azithromycin was found to reduce collagen-induced arthritis (CIA) in mice by inhibiting GRP78 activity (58). Thus, GRP78 is critical for the development of chronic arthritis.

When PERK dissociates from GRP78, it activates dimerization through autophosphorylation, leading to the phosphorylation of eukaryotic translation initiation factor 2α (eIF2α). eIF2α inhibits mRNA translation, suspends or attenuates protein synthesis, and alleviates the folding pressure of new and accumulated proteins in the ER. However, eIF2α only inhibits the majority of the protein translation process while promoting the synthesis of transcription factor 4 (ATF4) (59, 60). ATF4 positively feeds back to promote upstream GRP78 gene expression to accelerate the restoration of endoplasmic reticulum homeostasis and promotes NF-κB activation, which is involved in the synthesis and release of inflammatory factors (61). When ERS intensity increases and persists for a long period, sustained overexpression of ATF4 activates the nucleus-specific transcription factor (CHOP). CHOP is an important signaling molecule for the shift from survival to apoptosis in ERS, participating in Bcl-2/Bcl-Xl apoptosis regulation and promoting programmed cell death (61). In contrast to normal and osteoarthritis patients, GRP78 and p-eIF2α expression are increased in RA synovial tissues (57). Salubrinal, a selective eIF2α dephosphorylation inhibitor, has been shown to reduce the release of pro-inflammatory cytokines (IL-1β, IL-2, IL-13, and TNF) and the expression of the Dusp2 gene in a mouse model of CIA, alleviating arthritis progression (62, 63). The ERS inducer TG increased osteoclastogenesis through RANKL-induced NF-κB activation and ROS production, while IL-1β promoted RANKL-mediated osteoclast formation by upregulating GRP78, PERK, and IRE1. This process could be inhibited by 4-phenylbutyric acid (4-PBA) (64). Zinc ferrate nanoparticles and docosahexaenoic acid (DHA) have been reported to promote RA-FLS cell apoptosis by targeting the PERK-ATF4-CHOP pathway (65, 66).

ATF6 is an ER type II transmembrane protein. When ERS occurs, ATF6 dissociates from GRP78 and translocates to the Golgi apparatus, where its N-terminal portion is cleaved and activated. It then moves to the nucleus, where it binds to the heterodimer of the transcription factor XBP1, initiating the endoplasmic reticulum stress element (ERSE). This induces the expression of genes such as GRP94, GRP78, and ERP57, accelerating the degradation of intracellular unfolded proteins and reducing ERS (67). However, with continuous ERS stimulation, ATF6 induces the expression of apoptotic genes CHOP, promoting apoptosis (68). Studies on the mechanism of ATF6 in RA are relatively limited. High levels of ATF6 expression have been reported in RA synovium, and pro-inflammatory cytokines such as IL-1β and TNF can induce ATF6 expression in RA-FLS (57, 69). Tacrolimus, an important immunologic agent in RA treatment, reduces ATF6 expression and alleviates RA disease progression by inhibiting ERS-mediated osteoclastogenesis and inflammatory response (70).

IRE1 is a type I transmembrane protein on the ER with endonuclease activity. When IRE1 dissociates from GRP78, it undergoes oligomerization and auto-trans-phosphorylation to become IRE1α. This activates ribonucleic acid endonuclease, which shears X-box binding protein 1 (XBP1) mRNA. The sheared XBP1 mRNA then translocates to the nucleus, translating into the active transcription factor XBP1s. In the nucleus, XBP1s can induce the expression of the GRP78 gene, increase the degradation of endoplasmic reticulum-folded proteins, inhibit the synthesis of new proteins, and induce the transcription of the apoptosis gene CHOP (71). The IRE1α-XBP1s axis can splice to induce toll-like receptor-dependent cytokine secretion and maintain the autocrine cycle of FLS activation (72). Flavonoids from Litsea coreana were found to inhibit IRE1/mTORC1/TNF-α-regulated inflammatory responses triggered in peritoneal macrophages of CIA mice and ameliorated arthritis in a dose-dependent manner in CIA mice (73). In a study of peripheral blood mononuclear cells (PBMCs) from RA patients, it was found that the expression of GRP78, IRE1, and XBP1 was significantly increased in RA patients. Additionally, IRE1α activation in synovial macrophages was higher compared to healthy controls, whereas the progression of inflammatory arthritis in mice was prevented by knockdown of IRE1 (74). In exploring the mechanism of action of zinc ferrate nanoparticles on RA-FLS, the IRE1-XBP1 pathway was activated to induce apoptosis in RA-FLS, in addition to the activation of the PERK-ATF4-CHOP pathway (65). Moreover, IRE1 is a central regulator of B-cell differentiation, and both B-lymphocyte maturation and antibody plasma cell differentiation are regulated by the IRE1-XBP1 axis (75, 76).

This suggests that RA-FLS apoptosis resistance and the persistence of chronic inflammation are associated with ERS. Therefore, breaking this resistance relationship may be key to understanding the mechanism of RA.

## 4 Relationship between ERS and ferroptosis: a potential new direction for the treatment of RA

**Figure 3:**
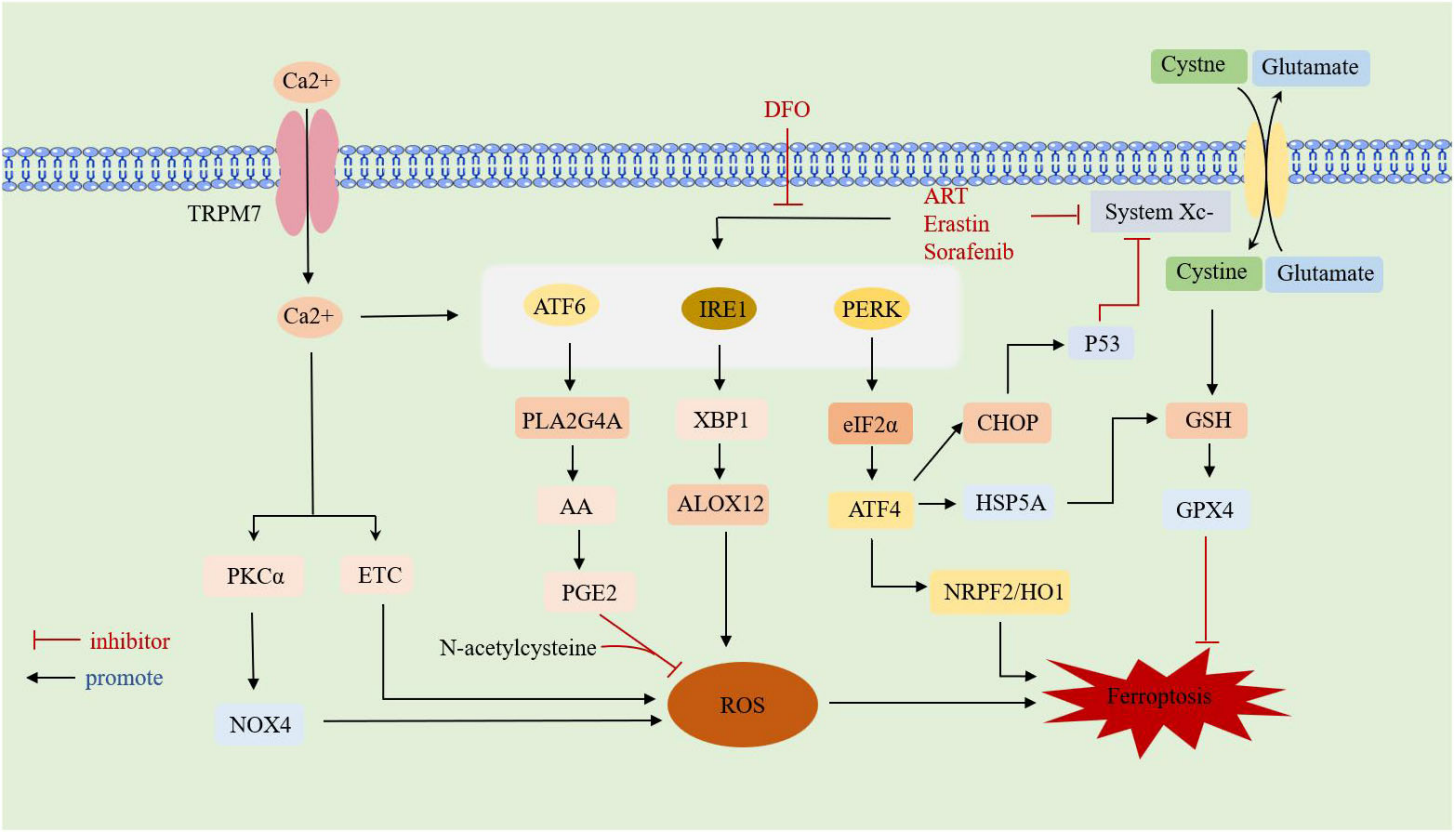
Overview of interaction between ferroptosis and ERS.TRPM7: transient receptor potential cation channel subfamily M member 7;DFO: deferoxamine; ART:artesunate;ATF: activating transcription factor; PERK: protein kinase RNA-like ER kinase; IRE1: inositol requirement protein 1; XBP1: homeostasis transcription factor X-box protein 1; eIF2α: eukaryotic translation initiation factor 2α; HSPA5: heat shock protein family A member 5; CHOP: C/EBP-homologous protein; ALOX12: arachidonate 12-lipoxygenase;GSH: glutathione; GPX4: glutathione peroxidase 4; HO-1: heme oxygenase-1; NRF2: nuclear factor-E2-related factor 2; PLA2G4A: phospholipase A2 group IVA;AA: arachidonic acid;PGE2: Prostaglandin E2;PKCa: calcium-activated protein kinase Cα;ETC: electron transport chain; NOX4: NADPH oxidoreductase; ROS: reactive oxygen species.

Studies have found that there is a shared pathway between ferroptosis and ERS, both of which are involved in cell survival processes. However, the mechanisms of ferroptosis and ERS in RA have only been studied separately, with no reports on their combined effects on RA. Therefore, the possible association between the two in RA can be explored through the review of studies on other diseases. An overview of interaction between ferroptosis and ERS is shown in (**Figure 3**).

It has been reported that Sorafenib inhibits the Xc- system transport of cysteine, leading not only to ferroptosis but also inducing ERS, such as eIF2α phosphorylation, and upregulation of ATF4 and cation transport-regulated homologue 1 (77). ATF4 has been suggested as an important regulator of SLC7A11 expression, and cells are more susceptible to drug-induced ferroptosis when ATF4 is knocked down (78, 79). The ferroptosis inducers ART and erastin both caused a time-dependent increase in GRP78 expression and induced ERS. Additionally, DFO inhibited ART-induced ERS (80). Yang J et al. (17) found that proteasome inhibitors could induce ERS and activate ferroptosis signals while studying the mechanism of drug resistance in liver cancer cells, causing tumor cells to adaptively tolerate ERS.

The activation of the PERK-eIF2α-ATF4-CHOP pathway mediated by ERS promotes p53 expression and is involved in the interaction between ferroptosis and apoptosis. p53, a classical tumor suppressor gene, inhibits the uptake of cystine by System Xc- through downregulation of SLC7A11 expression, leading to a decrease in the antioxidant capacity of cells. However, p53 is overexpressed in RA, where it regulates the balance between Th17 cells and Treg cells and accelerates chondrocyte destruction by blocking the cell proliferation cycle (80–82). Chen D et al. (78) found that ATF4 was significantly elevated in malignant gliomas. Activated ATF4 could transcribe System Xc-, promote GSH expression to elevate antioxidant capacity, and inhibit cellular ferroptosis, whereas knockdown of ATF4 caused ROS accumulation, inducing ferroptosis. Chen Y et al. (83) found that bisartemisinin (DHA), the active ingredient of the traditional Chinese medicine artemisinin, induced ferroptosis in glial cells. However, DHA itself induced ERS in glioma cells by activating PERK-ATF4-HSPA5, increasing the expression and activity of GPX4, thereby neutralizing DHA-induced lipid peroxidation and protecting glioma cells from ferroptosis. Thus, inhibiting the negative feedback pathway may be a therapeutic strategy to enhance the antitumor activity of DHA. In the study of Tagitinin C-induced ferroptosis in colorectal cancer cells, it was found that the mechanism of action of Tagitinin C might be related to the activation of the PERK-Nrf2-HO-1 signaling pathway induced by ER stress, leading to ferroptosis (16). Additionally, ATF4 can promote ferroptosis in tumor cells by increasing tumor microvessel generation and shaping vascular architecture in an SLC7A11-dependent manner (84). Subsequent studies have demonstrated that inhibition of PERK or ATF4 promotes ferroptosis in tumor cells by downregulating SLC7A11 expression, reducing GSH production, and thereby promoting ferroptosis (79, 85). For the cardiotoxic effects of sorafenib (SOR), the upregulation of ATF4 expression attenuated cardiomyocyte ferroptosis (15).

Increased ATF6α expression induced by androgen deprivation in prostate cancer renders prostate cancer (Pca) cells resistant to ferroptosis. This resistance is related to the ATF6α-PLA2G4A-mediated release of arachidonic acid (AA) and the resulting production of prostaglandin E2 (PGE2). PLA2G4A, a member of the cytoplasmic phospholipase family, catalyzes the hydrolysis of membrane phospholipids to release AA, which produces PGE2. Together with N-acetylcysteine, this process prevents heme-induced ferroptosis (86, 87). Studies on the progression of renal tubular epithelial mesenchymal transition (EMT) in diabetic nephropathy found that XBP1 overexpression increased HRD1 expression and inhibited NRF2 expression, enhancing cellular susceptibility to ferroptosis. This indicates that ERS triggers ferroptosis-associated EMT progression via the XBP1-HRD1-NRF2 pathway (14). Additionally, IRE1α-XBP1 induces the transcription of G protein alpha subunit 12 (ALOX12), promoting lipid peroxidation and mediating the onset of ferroptosis in hepatocytes (88).Various anti-solid tumor drugs can increase intracellular ROS and Ca^2+^, both of which can induce ERS. This is achieved by initiating Ca^2+^ channels on the tumor cell membrane, leading to the inward flow of extracellular Ca^2+^. When the concentration of Ca^2+^ in the cytoplasm reaches a certain level, the calcium storage pool in the endoplasmic reticulum also releases Ca^2+^, inducing ERS (89, 90). The accumulated ROS in cells interacts with Ca^2+^, increasing the permeability of the endoplasmic reticulum membrane by promoting redox modification of Ca^2+^ channels, leading to the release of Ca^2+^ reserves from the endoplasmic reticulum into the cytoplasm and inducing Ca^2+^ overload (91). Ca^2+^ overload regulates NADPH oxidase (NOX) and the electron transport chain (ETC) in mitochondria to promote ROS production (92). TRPM7, a transient membrane potential cationic protein channel with high permeability to Ca^2+^, is significantly expressed in RA-FLS cells. Inhibiting TRPM7 increases CHOP and calpain expression and increases apoptosis in RA-FLS cells *in vitro* (93). Notably, inhibition of TRPM7 also reduced articular cartilage destruction and suppressed chondrocyte ferroptosis in adjuvant arthritis rats (AAR). This is achieved by reducing intracellular Ca^2+^ overload through inhibition of TRPM7, which in turn reduces calcium-activated protein kinase Cα (PKCα)/NOX4 pathway-mediated ROS production, thus attenuating cellular ferroptosis and ERS (94, 95).

These results suggest that there are many interactions between ferroptosis and ERS. However, to determine whether these relationships also exist in RA, further more precise studies are needed to confirm their regulation and interactions in RA.

## 5 Discussion

This paper systematically outlines the current research on the mechanisms of ferroptosis in RA. As an iron-dependent form of cell death, ferroptosis involves three major mechanistic pathways, all contributing to RA pathogenesis. Notably, synovial fibroblasts, osteoblasts, osteoclasts, and immune cells exhibit varying sensitivities to ferroptosis. Therefore, exploring the underlying mechanisms in detail is crucial to understanding its role in RA. Additionally, the paper reviews the role of ERS in RA, highlighting that immune system overactivation, excessive autoantibody production, and apoptosis resistance in RA-FLS are linked to ERS-mediated UPR responses.

Moreover, with further research on the relationship between ferroptosis and ERS, studies in hepatocellular carcinoma, rectal carcinoma, glioma, and other tumors have identified a shared pathway between these processes. Ferroptosis can induce ERS and promote apoptosis through multiple pathways, while ERS can also increase ferroptosis sensitivity by interfering with lipid oxidation and antioxidants. However, further research is needed to determine whether the biological relationship between ferroptosis and ERS varies among different cell types and to reveal the mechanisms of death regulation for various cells. Additionally, it remains to be confirmed if the crosstalk between ferroptosis and ERS in RA aligns with research findings in other diseases.

In conclusion, ferroptosis is unlikely to function through a single pathway within the complex network of biological relationships. Consequently, based on the existing crosstalk between ferroptosis and ERS, it may become a new direction for future research, and may also become a new therapeutic target for RA.

## Author contributions

QA: Writing – original draft. HH: Writing – review & editing. YH: Writing – review & editing.
